# Assessing Sustainability in Cattle Silvopastoral Systems in the Mexican Tropics Using the SAFA Framework

**DOI:** 10.3390/ani11010109

**Published:** 2021-01-07

**Authors:** Fernanda Pérez-Lombardini, Karen F. Mancera, Gerardo Suzán, Julio Campo, Javier Solorio, Francisco Galindo

**Affiliations:** 1 Facultad de Medicina Veterinaria y Zootecnia, Universidad Nacional Autónoma de México, Av. Insurgentes Sur s/n, Ciudad Universitaria, México D.F 04510, Mexico; ferperlom@gmail.com (F.P.-L.); kfmancera17@gmail.com (K.F.M.); gerardosuz@unam.mx (G.S.); 2 Instituto de Ecología, Universidad Nacional Autónoma de México, Av. Insurgentes Sur s/n, Ciudad Universitaria, México D.F 04510, Mexico; jcampo@ecologia.unam.mx; 3 Facultad de Medicina Veterinaria y Zootecnia, Universidad Autónoma de Yucatán, Carretera Mérida-Xmatkuil Km. 15.5 Tizapán, Mérida 97100, Mexico; ssolorio@correo.uady.mx

**Keywords:** sustainability, cattle, SAFA, tropics, native silvopastoral, intensive silvopastoral

## Abstract

**Simple Summary:**

Cattle production is associated with deforestation in tropical Mexico. Silvopastoral systems have been proposed as a feasible alternative for sustainable livestock production and knowledge on their advantages and areas of opportunity, compared to traditional pasture paddocks, is needed for their implementation. This work compares three types of production systems, native and intensive silvopastoral systems and monoculture systems in Yucatán, Mexico, using the Sustainability Assessment for Food and Agriculture (SAFA), which evaluates sustainability in different themes and dimensions. With SAFA, visual representations of overall sustainability or polygons were obtained. Additionally, percentages of SAFA themes positively and negatively valuated were calculated. Native farms had positive ratings for themes related to environmental integrity and Social Well-Being, whereas intensive excelled on Holistic Management. SAFA identified native systems as an option for sustainable production; however, areas of opportunity in all farms were discovered in every dimension. This is the first comparative study using SAFA to evaluate differences in farming systems in the Mexican tropics, and it provides valuable information to generate policies and incentives for sustainable silvopastoral production, as well as to develop new evaluation tools that are more appropriate for this region.

**Abstract:**

The sub-humid native rainforest in Yucatan is one of the most endangered in Mexico. Cattle production is one of the main causes of land use change and silvopastoral systems are a feasible alternative. This work compares the sustainable performance of silvopastoral (native and intensive) and monoculture cattle farms in the state of Yucatan using the Sustainability Assessment for Food and Agriculture (SAFA) framework. Questionnaires and semi-structured interviews were applied in 9 farms. Responses were fed to the SAFA Tool to obtain sustainability polygons. Percentages of SAFA themes positively and negatively valuated were calculated. Native farms had positive ratings for Participation, Land, Biodiversity and Cultural Diversity, whereas intensive excelled on Holistic Management. Native farms had limited ratings for Decent Livelihood. Native farms (and one intensive silvopastoral farm) had the highest percentages of themes positively valuated compared to monocultures (and one intensive silvopastoral farm), which scored the lowest. Positive evaluations identified native systems as an option for sustainable production; however, areas of opportunity in all farms were discovered. This is the first comparative study using SAFA to evaluate differences in farming systems in the Mexican tropics, providing valuable information to generate policies and incentives on sustainable livestock production, as well as for improving evaluation tools for local application.

## 1. Introduction

Tropical deforestation to obtain grazing paddocks has a great impact on biodiversity and environmental service. In addition to land conversion, global population will increase to 9600–10,000 million in 2050 [1], as well as the demand of meat and milk by 73% and 58% respectively, compared to 2010 [2,3]. In 2011, 21% of Latin America lived in rural areas, where livestock represents food sources and means to improve their economic and social situation [4]. In Mexico, most cattle grazing occurs in tropical regions including the state of Yucatan, which converted 310,000 ha of tropical forest to pastures between 2001 and 2014 [5]. Therefore, it is necessary to find new strategies to guarantee food security in rural areas, methods to increase animal production, and strategies to mitigate climate change. Silvopastoral systems (cattle raised in paddocks associated with trees and shrubs) are an alternative as they associate with increased photosynthetic rates, nitrogen fixation, nutrient recycling, biomass production and organic matter in soil [6]. These also promote better animal welfare and the continuation of environmental services, such as carbon sequestration, water preservation, soil rehabilitation and biodiversity conservation [7].

Intensified Silvopastoral Systems (IS) integrate technical knowledge, such as the inclusion of specific plant species. Meat production in IS can be 12 times higher than in extensive systems and 4.5 times higher than systems with improved pastures [8]. Likewise, methane emissions/tonne of meat/year are 1.8 times lower that in extensive cattle systems [9]. In contrast traditional or Native Silvopastoral Systems (NS) are not actively managed but are more likely to include native flora and fauna. NS and IS are alternatives to Monoculture Systems (MS), which are intrinsically related to a reduction of ecosystem services [10].

To foster the implementation and preservation of silvopastoral systems, it is necessary to generate information on the strengths, weaknesses and opportunities associated, as well as the differences among NS, IS and MS [11]. This information can be gathered using evaluation systems designed to estimate the sustainability performance of farms, such as the Sustainability Assessment for Food and Agriculture (SAFA), designed by United Nations’ Food and Agriculture Organization (FAO). SAFA main goal is the assessment of organizations through the evaluation of components of the supply chain. It covers environmental, social, economic and governance dimensions. Some of its most important purposes are self-assessment to stimulate learning and improve management, planning and legislation development and monitoring of project outcomes [12].

The SAFA framework has been previously used for several purposes, such as measuring the sustainability performance of organic farms in Italy and Saudi Arabia [13], or smallholding coffee farmers in Colombia [14]. Similarly, this tool was used to compare five types of agricultural systems, determining that all systems, excluding agribusiness, where similar to each other and had good performance in all sustainability dimensions [15]. SAFA was also useful to establish the differences between organic and conventional banana crop systems, showing that organic performed better on the governance, environmental and economic dimensions, whereas the conventional systems were better scored on the social dimension due to their size and processes [16]. SAFA has also been used to establish interventions; when applied in two Amazonian communities in Ecuador, critical areas, such as atmosphere, animal welfare and corporate ethics, were identified as critical to be strengthened with the use of communication and information technologies [17]. Likewise, in Cambodia, SAFA helped determine the success of training on smallholders, demonstrating that trained farmers increased their net income, had more diverse food production and had more planning aimed to mitigate their impact on the environment [18].

SAFA has also been applied in the evaluation of sustainability performance in cattle farming systems. For instance, Gayatri et al. assessed the sustainability performance of three types of management systems present in cattle farms in Indonesia: managed only with family labor, managed only with hired labor and managed with hired labor and the head of the family working as the middleman in the local marketing system. It was found that farming systems with more resources and hired labor, and with the household head working as a middleman had better sustainability scores that those managed only by family members and with fewer resources, indicating that there is a great need to increase sustainable practices in farms managed only by family members [19]. Hanisch et al. [20] used the SAFA Smallholders App, developed to further expand the applicability and implementation of the SAFA Guidelines. They identified strategies to intensify pasture use and increase animal productivity in traditional and improved silvopastoral systems, composed by erva-mate and araucaria seed extraction and cattle rearing in the Araucaria forests in Brazil. While these examples have used SAFA to evaluate differences in the sustainable performance of cattle farming and in silvopastoral systems, this tool needs to be further validated under a diversity of conditions and in the presence of different landscape structures [21], as it could be used to promote the transition and implementation of systems such as silvopastorals.

Although several studies on silvopastoralism as an approach to sustainability exist [22,23,24], these systems have not been successfully adopted in Yucatan. Furthermore, there are no data on the features of these systems in the economic, social and environmental dimension, which are paramount to understanding and successfully implementing them. Therefore, this study contrasted the existing cattle farming systems in Yucatan (NS, IS and MS). To date, this is the first comparative study using a multicriteria sustainability assessment tool, such as SAFA, to evaluate fundamental differences in farming systems and identify the necessary criteria to properly promote cattle farming of low environmental impact in Mexico. We aim to provide valuable and urgent information to generate policies and incentives on sustainable livestock production in Mexico, as well as for improving evaluation tools for a more local and accurate application. Our objective is that the results obtained allow the identification of possible strategies to improve sustainability performance, as well as to evaluate the utility of the SAFA framework in the Mexican tropics. Our hypothesis is that NS and IS will have a better sustainability performance than MS, with emphasis in the environmental dimension, due to the benefits that silvopastoral systems provide to animal welfare, wildlife diversity and soil quality.

## 2. Materials and Methods

This study was conducted in the state of Yucatan, Mexico (20°24′1.5012″ N, 89°8′5.4852 W). Average annual temperatures in the state range 24–28 °C, with an average maximum temperature of 36 °C and average minimum of 16 °C. The lowest precipitation values in the state are 500 mm, and the highest range from 1200 to 1500 mm [25]. The nine farms evaluated belonged to three municipalities: Tzucacab, Merida and Tizimin, that were chosen according to their type of predominant land use and their native vegetation coverage. Tzucacab was the municipality with the widest coverage of native vegetation; Mérida the one with the biggest urban area and a medium native vegetation coverage; and finally, Tizimin with half of its territory covered with pastureland. More details on municipalities can be found in Pérez-Lombardini [26]. In each municipality, three farms were chosen to represent one of the following production systems:

- Native Silvopastoral (NS) system: pastures with unmanaged native shrubs and trees (Gómez-Cifuentes et al., 2019). The NS farms were: Roble in Tzucacab (NS1); Santa Teresa in Merida (NS2); Xhopel in Tizimin (NS3)
- Intensive Silvopastoral System (IS): integration of fodder shrubs at high densities (>10,000 plants ha−1), productive pastures and trees (Murgueitio et al., 2011). The IS farms were: Kakalnah in Tzucacab (IS1); Kampepem in Merida (IS2); Golondrinas in Tizimin (IS3)
- Monoculture System (MS): conventional grazing system based on monoculture of grass (Mancera et al., 2018). The IS farms were: Ramonal in Tzucacab (M1); UADY in Merida (M2); Escalera in Tizimin (M3)

From the farms selected, NS1, IS2 and MS2 were double-purpose cattle systems (production of milk and meat) and the rest were meat production systems. All farms belonged to smallholders working with limited resources in rural areas.

### 2.1. Description of SAFA Methodological Framework

A complete explanation of SAFA can be found in the SAFA Guidelines version 3.0 [27]. SAFA integrates four sustainability dimensions: Good Governance, Environmental Integrity, Economic Resilience and Social Well-being. These dimensions are divided into 21 themes, 58 subthemes and 116 indicators for evaluation. The assessment follows four stages: mapping, contextualization, selection of tools and indicators and reporting. Mapping consist of the use of organizational documents, value chain maps and a detailed description of the farms to define assessment goals and identify the boundaries of the elements included in the assessment. The relevant sustainability subthemes in the farm are recognized. Each subtheme has default indicators determined by SAFA aimed to identify measurable criteria for sustainable performance. Indicators fall into five possible ratings ranging from 0 to 100%, according to the farm performance: best (80–100%), good (60–80%), moderate (40–60%), limited (20–40%) and unacceptable (0–20%). The characteristics of the “best” and “unacceptable” ratings are already defined by SAFA; hence, contextualization focuses on determining the characteristics of the intermediate ratings for indicators, using the geographical, regional, climatic and socioeconomic information gathered during mapping. At this stage, it is possible to omit or add specific themes when deemed necessary and identify replacements for default indicators. When the assessor or smallholder is unable to respond to some indicators or themes, these are omitted with justification. The selection of tools and indicators initiates with the data collection process, where the appropriate measurement tools based on budget, availability and the default indicators to be assessed are chosen. Likewise, an accuracy score is calculated considering timeframe (if the data is sourced from the most current information), type (if the data is primary, secondary or an estimation) and methodology (if data was collected using the SAFA guidelines). At this stage, indicators are measured within the 5 possible ratings and, particularly in the environmental dimension, indicators are divided into 3 types: performance-based indicators (results of compliance with an objective with the ability to identify trends and communicate results), practice-based indicators (the presence of tools and systems to enable best practices) or target-based indicators (the presence of plans to reach targets). Their use is determined by the conditions of the production unit and preference is given to performance indicators. Once indicators are measured, the SAFA tool version 2.1.50 is fed to complete the mapping, contextualization and indicators stages and produce SAFA sustainability polygons and final reports as part of the reporting stage. Polygons are aimed to synthetize and facilitate visualization or results. Definitions of dimensions and themes are summarized in Table 1.

### 2.2. Theme and Subtheme Exclusion

According to the SAFA Framework, if a performance indicator is omitted because of lack of available data or other reasons, the omission must be justified and the omitted indicator may be considered excluded from both the sub-theme rating and the total possible sub-theme accuracy score. For this evaluation, five subthemes were excluded during the contextualization stage:

- Subtheme: Stability of production. Theme: Vulnerability; Dimension: Economic Resilience. The units evaluated here work as their own providers by producing their own forage.
- Subtheme: Air quality. Theme: Atmosphere; Dimension: Environmental Integrity. Farms did not have formal plans to reduce air contaminants nor considered it a specific goal and it was unfeasible to measure standard pollution values.
- Subtheme: Water quality. Theme: Water; Dimension: Environmental Integrity. Empirical measures to preserve water quality are unfeasible and residual water samples were not possible to obtain.
- Subtheme: Material use. Theme: Materials and Energy; Dimension: Environmental Integrity. The use of construction material is minimal and the use of materials to calculate nutrient balance was unfeasible to obtain due to the lack of records.
- Subtheme: Product information. Theme: Product Quality and Information; Dimension: Economic Resilience. The final product of these farms is calves. Traceability of calves after sell was impossible under the Mexican trading rural system.

### 2.3. Evaluation of Indicators

Considering the 116 indicators stablished by SAFA, four questionnaires were formulated to measure indicators:

- Questionnaire A was applied to the unit manager and was composed of 164 questions and eleven sections: productive activity, infrastructure, animal inventory, reproductive and productive indicators, calf handling, herd handling, health control, feeding, paddock/pen characteristics, operational costs, and soil characteristics. This questionnaire was applied once in each farm (n = 9)
- Questionnaire B was also applied to unit managers and comprised 12 questions addressing: mission statement, number of employees, working conditions and benefits, characteristics of employment, health and safety plans, emergency procedures and training. This questionnaire was applied once in each farm (n = 9)
- Questionnaire C was applied to 30 farm workers and comprised 66 questions divided in two sections. Section I included questions about gender, age, years working for the farm, work situation (permanent or temporal worker) and highest level of education completed. Section II included questions on biological contaminants, work schedule and environment, training and development, health risks related to their job, work organization and working rights, legislation, leadership style and participation and job position and salary.
- Questionnaire D was applied to members of the local community where farms were located and included 30 questions on the following topics: commitment to sustainability-related themes, commitment with the community and citizen participation, health and security in relation to the farm, relationship between the farm and the community and animal welfare. This questionnaire was applied to a total of 290 subjects. The number of questionnaires applied to the local communities where farms were located were: NS1 = 21, IS1 = 63, MS1 = 63, NS2 = 38, IS2 = 44, MS2 = 12, NS3 = 25, IS3 = 51, MS3 = 36.

Questionnaires were applied during 2 months in the rainy season (July and August 2015). During this period, two visits were made to the farms. On the first visit, 3–5 days per farm were occupied to characterize each unit and apply questionnaires A, B and C. The second visit was to apply questionnaire D. The information obtained was used to feed the application SAFA tool version 2.1.50, producing the corresponding SAFA sustainability polygons further presented in the Results section. The application of questionnaires and overall work was revised and approved by the CICUA committee as part of the procedures applied to postgraduate research undertaken in the Faculty of Veterinary Medicine at the National Autonomous University of Mexico.

In addition to questionnaires, to evaluate the theme Animal Welfare of the Environmental Integrity dimension, the Welfare Quality^®^ (WQ) protocol for the evaluation of dairy cattle [28] was used. WQ is referred to by other sustainability assessment protocols [29] and is recognized as a multidimensional, animal-based welfare assessment tool. The SAFA indicators of the Animal Welfare theme were estimated with the WQ protocol as follows [28]:

- Indicator Animal Health Practices, subtheme Animal Health = Good Feeding principle which includes the evaluation of:
  ○ Body condition
  ○ Water provision
- Indicator Animal Health, subtheme Animal Health = Good Health principle which includes the evaluation of:
  ○ Lameness.
  ○ Integument alterations (injuries, inflammation, and alopecia)
  ○ Presence or absence of each health indicator (nasal discharge, ocular discharge, hampered respiration, diarrhea, vulvar discharge and ectoparasites)
  ○ Coughing and sneezing
  ○ Disbudding/dehorning, tail docking
- Indicator Humane Animal Handling Practices; subtheme Freedom of stress = combination of the following criteria:
  ○ Body condition
  ○ Water
  ○ Cleanliness of udder, flank/upper legs and lower legs
  ○ Time needed to lie down in pens: Focal observations of time
  ○ Lameness
  ○ Integument alterations (injuries, inflammation and alopecia)
  ○ Presence or absence of each health indicator
  ○ Coughing and sneezing
  ○ Disbudding/dehorning, tail docking
  ○ Agonistic behaviors
  ○ Avoidance distance
- Indicator Appropriate Animal Husbandry; subtheme Freedom of Stress = Appropriate Behavior principle, which includes the evaluation of:
  ○ Agonistic behaviors
  ○ Access to pasture: hours of the day herd spent in the paddock
  ○ Qualitative behavior assessment (observation of herds with assignation of an emotional state previously standardized by the observer (active, relaxed, uncomfortable, nervous, happy, etc.)
  ○ Avoidance distance
- Indicator Freedom of Stress; subtheme Freedom of Stress = final farm score obtained with the combination of all criteria

Obtained data was processed with the software program Welfare Quality^®^ scoring system [30]. The scoring values obtained with the Welfare Quality^®^ protocol fall in values from 0 to 100; therefore, values obtained with this protocol were directly assigned to the corresponding SAFA rating. More details on the evaluation of each indicator can be find in the Welfare Quality^®^ assessment protocol for cattle [28].

To evaluate the indicator “Genetic Diversity in Wild Species” (subtheme Genetic Diversity; Theme Biodiversity: dimension Environmental Integrity) direct wildlife monitoring was used. Capture-recapture of birds, bats and small rodents was performed in the farms during the same period. Bats and birds were captured using mist nets, whereas Sherman traps were used for small rodents. With this information, abundance (number of individuals) and species richness (number of species) were calculated for each farm. Afterwards, the Shannon–Wiener diversity index (H’) was calculated for each animal group. Once diversity indexes were obtained for each farm, they were reinterpreted as percentages assigning the percentual value of 100% to the greatest index obtained. The percentages calculated were used to assign SAFA ratings. Additionally, to address the biodiversity indicator Locally Adapted Varieties/Breeds (subtheme Genetic Diversity; Theme Biodiversity; Dimension Environmental Integrity), the total number of endemic species (birds, bats and small rodents) encountered in each farm was counted. SAFA ratings were assigned as follows: best = 5 spp; good = 4 spp; moderate = 3 spp; limited = 2 spp; unacceptable = 1 spp.

To evaluate the indicator Diversity and Abundance of Key Species (subtheme Species Diversity; Theme Biodiversity; Dimension Environmental Integrity), calculations considering if species were endemic or introduced and if they were classified as “at risk” by the Official Mexican regulation NOM-059-SEMARNAT-2010 [31] were performed. Details on these calculations can be found in Pérez-Lombardini [26]. The values obtained with these calculations were only considered in order to obtain a percentage that integrated both, number of species and additional features, and that could be assigned a SAFA rating.

To evaluate the indicators related to the subtheme Soil Quality (Theme Land; Dimension Environmental Integrity), values obtained through the direct measurements of soil properties were used. Soil characteristics evaluated for each indicator are summarized in Table 2. A detailed methodology for the measurement of these soil characteristics is described by Alvarado-Figueroa [32]. Using measurements obtained in undisturbed soils in the same areas as reference maximum values, the values obtained in ranches were converted into percentages to allocate SAFA ratings.

### 2.4. Graph Interpretation for Dominant Dimension and Sustainability Performance

SAFA provides a polygon of a production unit that can only be visually interpreted and compared with other farms. Therefore, to better identify farms with better or worse sustainability performance, the integrated procedure was as followed:

I. For each farm, the percentage of themes rated as best, good, moderate, limited, and unacceptable were calculated by counting the number of themes per rating classification and considering the total number of themes evaluated in each farm (21) as 100%
II. Percentages of themes in each rating category were later labeled as “positive” (best and good rating percentages) and “negative” (limited and unacceptable rating percentages). The themes found at the category “moderate” were eliminated as they did not provide relevant information for the interpretation
III. Percentages of themes in rating categories labeled as “positive” and “negative” were added up, resulting in percentages of “positive valuations”, and “negative valuations”
IV. Once calculated, farms were listed according to the calculated percentages of positive valuations: the highest positive valuation percentage was assigned to the top of the list while the lowest to the bottom. If two farms had the same percentage of positive valuation, the highest ranked was the one with the lowest percentage of negative valuation
V. Positive and negative valuation percentages were rated according to the SAFA system (best = dark green; good = light green; moderate = yellow; limited = orange; and unacceptable = red). To assign classification colors, the highest percentage of positive valuations was divided by five to obtain the lowest possible percentage which was deemed as “unacceptable”. The values for the rest of the ratings were obtained by multiplying the minimum value by the factors 2 (limited), 3 (moderate), 4 (good) and 5 (best). Percentages were rounded down when decimals were ≤0.5 and rounded up when decimals ≥0.6.
VI. For negative valuations, the highest percentage of negative valuations was divided by five, to obtain the lowest possible percentage which was deemed as “best”. The values for the rest of the ratings were obtained by multiplying the minimum value by the factors 2 (good), 3 (moderate), 4 (limited) and 5 (unacceptable) factors. Percentages were rounded down when decimals were ≤0.5 and rounded up when decimals ≥0.6.

**Figure 1:**
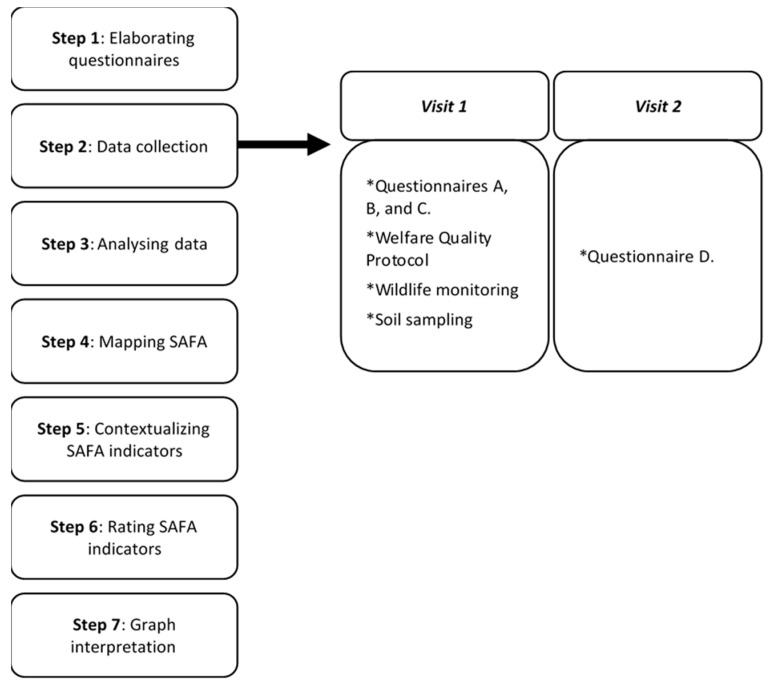
Timeline representing the procedure of the SAFA (Sustainability Assessment of Food and Agriculture Systems) methodological application for the construction of the sustainability polygons.

A flux diagram summarizing the methodological application of the SAFA framework is present in Figure 1:

## 3. Results

**Figure 2:**
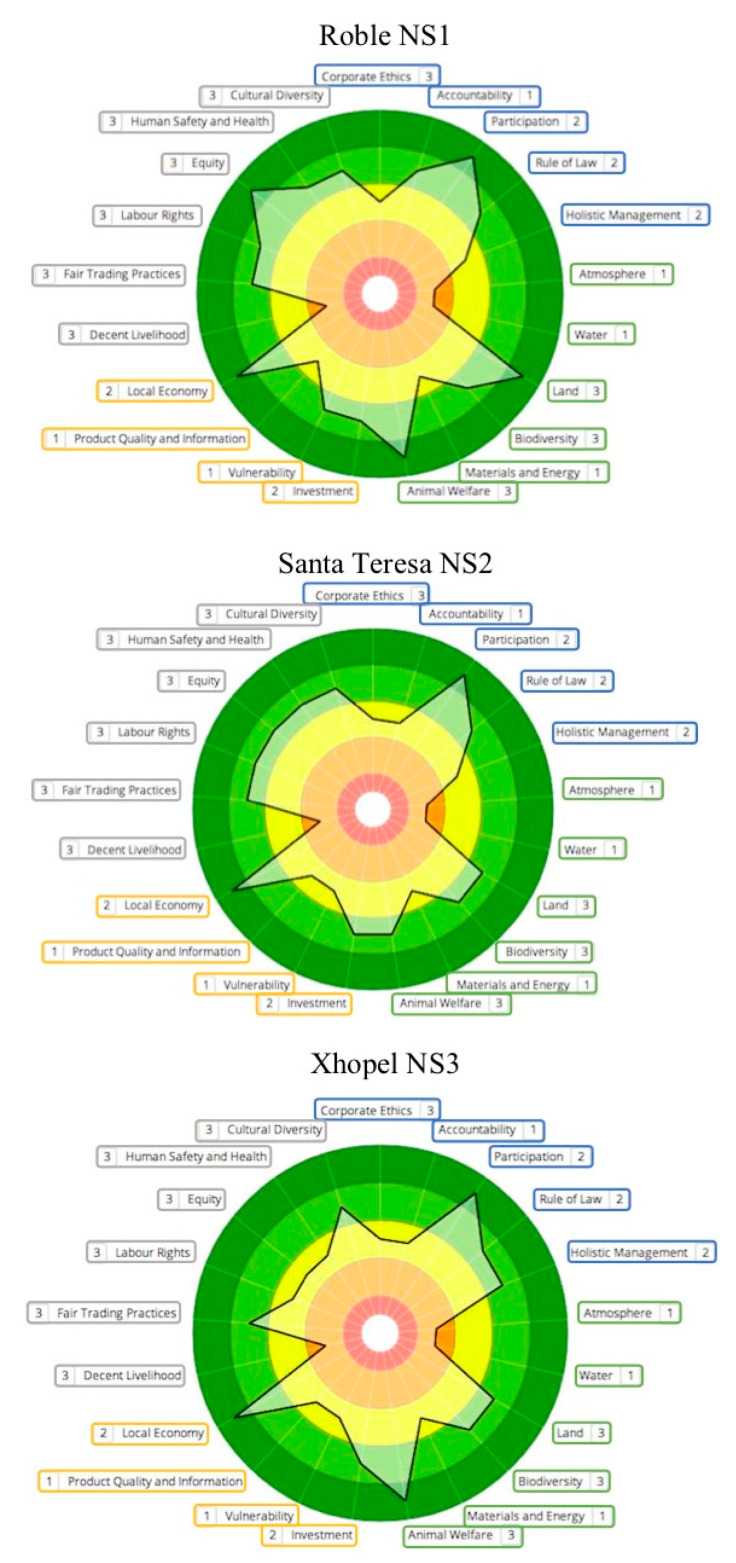
Sustainability polygons for farms representing Native Silvopastoral systems (NS).

**Figure 3:**
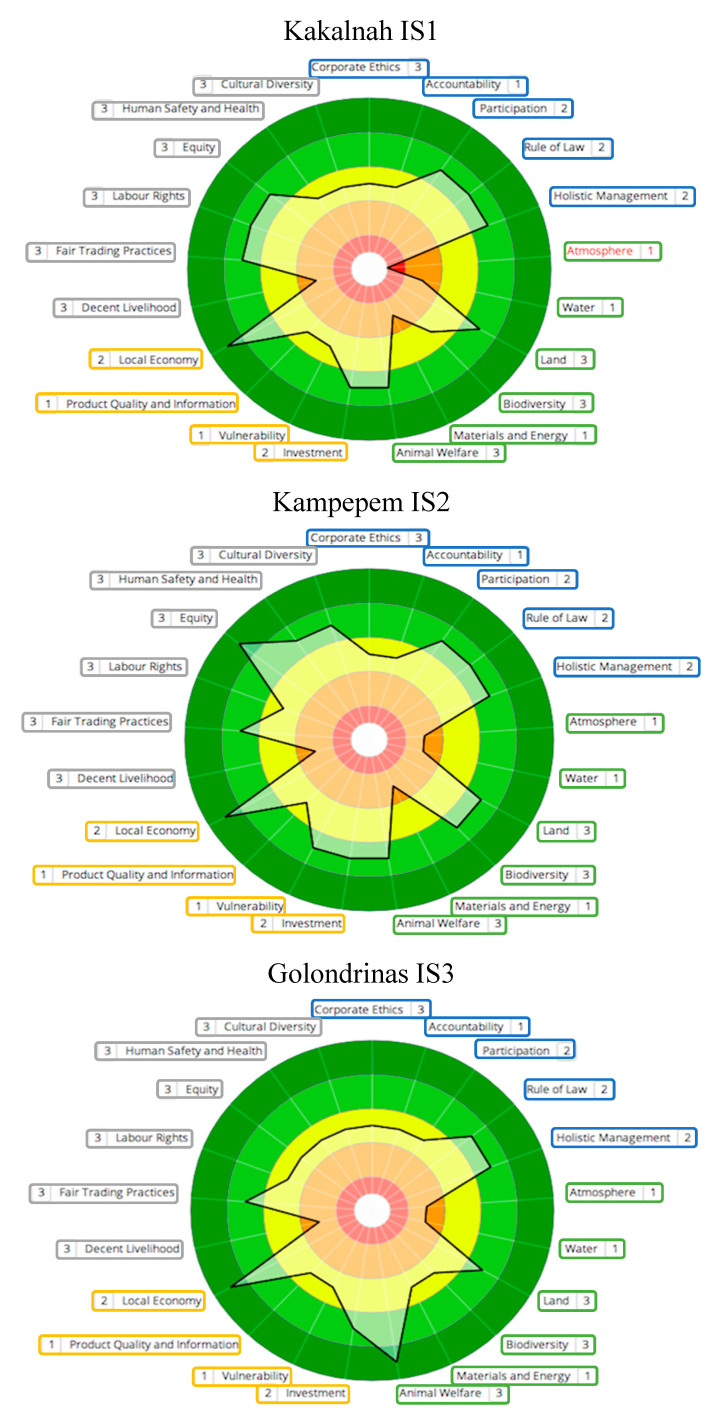
Sustainability polygons for farms representing Intensive Silvopastoral systems (IS).

**Figure 4:**
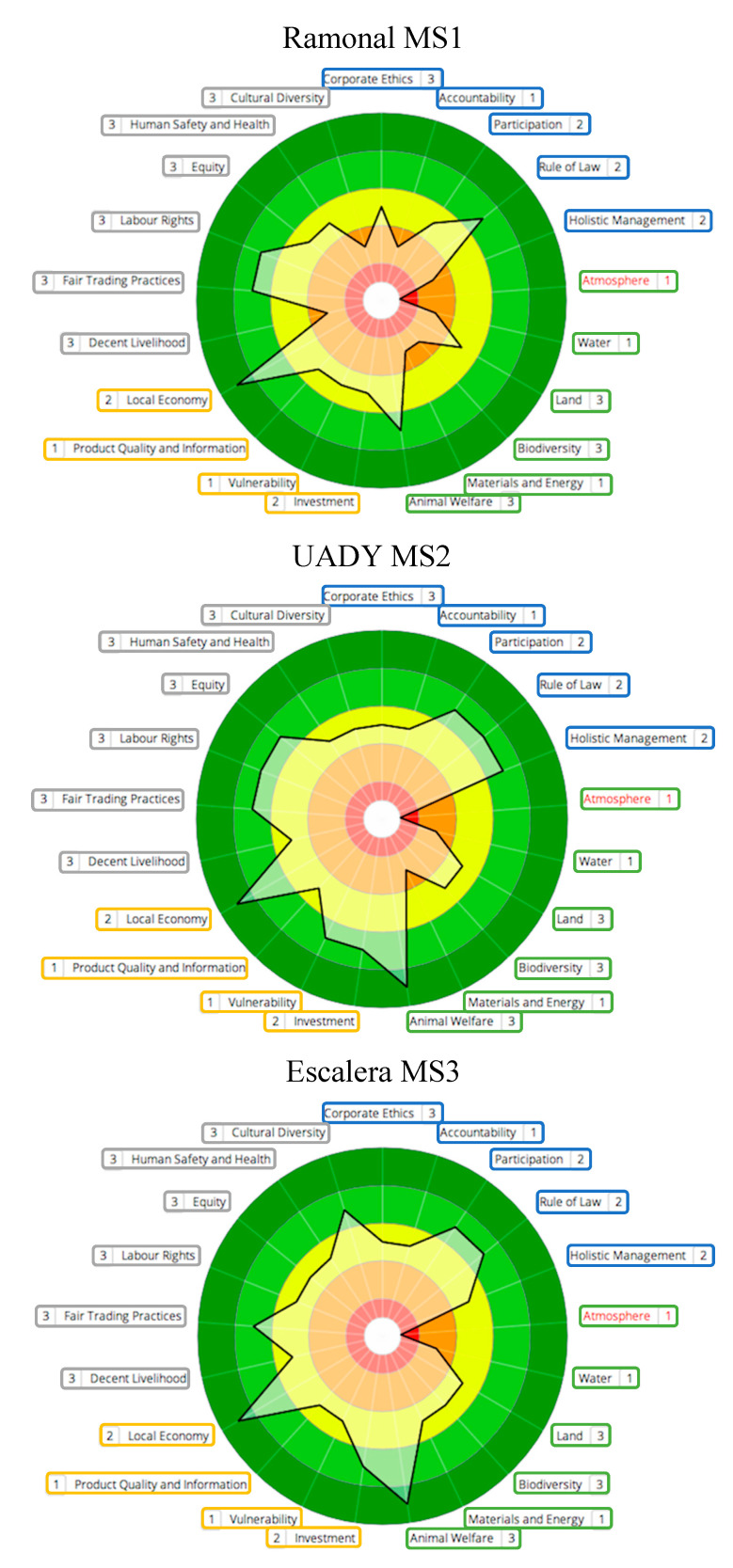
Sustainability polygons for farms representing Monoculture systems (MS).

Sustainability polygons are shown in Figure 2 (NS systems), Figure 3 (IS systems) and Figure 4 (MS systems). Circle colors represent ratings determined by SAFA (best = dark green; good = light green; moderate = yellow; limited = orange; red = unacceptable). Colored boxes divide themes by dimensions (good governance = blue; environmental integrity = green; economic resilience = yellow; social well-being = grey). The thick black line connects theme performance following the rating obtained. Numbers next to each theme represent the quality of the information used for indicators evaluation (1 = high quality; 2 = medium quality; 3 = low quality). Based on the information provided by SAFA polygons, an analysis of SAFA dimensions and themes was performed.

### 3.1. Good Governance

Farms obtained moderate ratings for Participation when owners made decisions alone (IS3) or when poor communication between owners and employees was prevalent (MS1). In contrast, NS were managed directly by family and/or long-term workers who were included in the decision-making process and were rated as best. For Holistic Management, all IS farms, NS3 and M2 were rated as good, as they considered economic, social and environmental impacts to perform integral evaluations of their performance, in contrast with other farms. For Accountability, most farms lacked explicit communication about the unit performance to all workers and were rated as moderate. Likewise, all farms we also rated as moderate for Corporate Ethics, as risk analyses and clarity on the betterment of sustainable performance were needed. For Rule of Law, all farms rated good.

### 3.2. Environmental Integrity

For Land, all M farms have the least amount of practices for soil conservation and rated as moderate; meanwhile, all IS, NS2 and NS3 farms were rated as good, with NS1 rated as best. NS farms and IS2 had the highest proportion of live fences and fruit trees and had good Biodiversity ratings. NS systems had limited paddock manipulation (i.e., clearing, burning, etc.) and IS2 included sustainable practices, such as diversified production through timber-yielding trees, and preservation of tolchés (semi-conserved forest areas). In contrast, M1 presented undiversified production, agrochemical use, and no species conservation goals and rated as limited. For atmosphere, all M units and IS1 had large amounts of machinery and presented few practices to improve air quality, thus rated as unacceptable. For the rest of the farms clear goals to reduce air pollutants or GHG release were absent and they were rated as limited. Likewise, for the theme Water, all units lacked clear goals to decrease water pollution or increase water efficiency and were rated as limited. Finally, for Animal Welfare, all farms were rated as best or good.

### 3.3. Economic Resilience

In this dimension, all farms bought local products for their farm and sold their own products locally, thus rated as best for the theme Local Economy. The second-best rated theme was Investment, where all farms (except M3) presented good ratings, as they had sustainability goals and made investments to achieve them such as donations to the neighboring communities. M3 lacked investment records, plans and financial resources. For Vulnerability, only NS1, IS2 and M2 had greater production diversity and good ratings. All other farms were rated as moderate because they were not present in different kinds of markets and lacked diversification. For the theme Product Quality and Information, all farms were rated as moderate, as the product (calves), even when having a good sanitary management, lacked traceability due to the production conditions in Tropical Mexico.

### 3.4. Social Well-Being

For Decent Livelihood, all NS and IS farms, and MS1, were rated as limited whereas the rest as moderate, as workers receive limited support in terms of training and fair salary. For Human Safety and Health farms NS1, NS2 and IS2 were rated best, and the rest as moderate, with best rated farms presenting the best working conditions and a clean and healthy environment. In the theme Labor Rights, farms NS1, NS2, IS1, M1 and M2 were rated as good and the rest as moderate; however, despite the good ratings, none of them had full compliance of benefits for their employees, as only basic ones, such as medical insurance, where given. For Equity, farms NS1 and IS2 were rated as best, whereas IS1, NS2 and M2 had good ratings and the rest, moderate. Best and good rated farms presented no impartiality between genders, age groups, and family and non-family members, as well as support for vulnerable groups. Moderate rated farms presented discrimination from owners to workers and among employees, calling for improvements in this area.

For Cultural Diversity, all NS farms as well as M3 and IS2 were rated as good, whereas IS3, IS1 and M2 were rated as moderate and M1 as limited. Farms rated as Good respected cultural variety, with emphasis in Mayans, the prevalent indigenous group in the area. Moderate rated farms did not promote emphatically indigenous values, but did not present discrimination, which M1 did, by identifying some workers as “mayeros”, a derogatory name used for Mayan persons that cannot speak Spanish fluidly. Finally, for Fair Trading all farms were rated as good, as calf prices were reported as stable for the last years and are not set by buyers, contributing to market fairness.

### 3.5. Sustainability Performance of Farms and Utility of SAFA Framework for the Evaluation of Silvopastoral Systems

Table 3 shows the overall sustainability performance of the farms analyzed. Colors represents ratings determined by SAFA (best = dark green; good = light green; moderate = orange; limited = yellow; red = unacceptable). Percentual ranges calculated for rating assignation of positive valuations: best = 55–67%; good = 41–54%; moderate = 28–40%; limited = 14–27%; unacceptable = 0–13%. Percentual ranges calculated for rating assignation of negative valuations: best = 0–8%; good = 9–15% moderate = 16–23%; limited = 24–30%; unacceptable = 31–38%. NS = Native Silvopastoral; IS = Intensive Silvopastoral; M = Monoculture.

## 4. Discussion

The results showed that NS had positive ratings for Participation, Land, Biodiversity and Cultural Diversity, and IS farms for Holistic Management. NS had limited ratings for Decent Livelihood, due to poor training and underpay. Positive evaluations identified NS as an option for sustainable production; however, it was also recognized that group participation, environmental knowledge and awareness, identifications of potential business risks, technical support and training were key factors to improve sustainability in all farms.

In this sense, the criteria good Participation suggests that family and long-term workers are playing a positive role in silvopastoral practices. Equally, IS structure and vegetal composition naturally imply an increased all-inclusive administration that was reflected in good ratings for Holistic Management. The benefits of endemic trees and shrubs present in NS systems, such as improved soil fertility and the reduced use of chemicals which favored the theme Land, were outstanding and intimately related to the ability of silvopastoral systems to remove greenhouse gases from the atmosphere. Nonetheless, aspects such as technical support, increased knowledge and awareness on environmental services were identified as key factor for increasing the disposition and engagement of farmers on themes such as Water and Atmosphere.

It is also worth mentioning that silvopastoral systems, native or intensified, are in fact, more complex than a mere classification in terms of intensification. For instance, these systems can be composed of live fences, scattered trees, riparian forest or forest fragments or a combination of all. Presenting varied percentages of tree and shrub coverage can provide different environmental and animal welfare benefits [24], thus yielding varied sustainability performance. Silvopastoral systems can also be more dynamic and heterogenic than monocultures, and their structure and composition may remain or change over time [33]. Hence, their sustainability performance and assessment may differ at different time points, adding complexity to its evaluation.

The dimension Good Governance seeks for a sustainable-oriented governance structure and compliance with all the themes is not an easy task. According to the visual analysis of polygons, two themes within this dimension appeared to be influenced by NS: Participation and Holistic Management. High ratings for Participation in NS can be related to the involvement of family members in farm management. It has been observed that silvopastoral farmers have strong ties to the land (39%), a strong personal motivation to improve their farm (25%) and satisfaction from the learning experience in silvopastoral systems (46%) [34]. Worker satisfaction increases long-term employment and Participation, thus encouraging farmers to contribute with the farm development and to adopt new technologies and practices [8,19,35].

Although Holistic Management and Accountability are perceived as expensive by smallholders due to the requirement of holistic audits and full cost accounting [36], IS in this study achieved good ratings. Since silvopastoral intensification requires continuous development of producers’ capabilities and stakeholders’ co-responsibilities [10], it is possible that the inherent demand of improved resource management in IS resulted in better performance on this theme. As holistic accounting and management are many times performed by facilitators such as NGO’s and farmer associations [36], engaging farmers with these groups could help improve the score on these themes for all systems.

Group work was perceived as important for Accountability and Corporate Ethics, as most farms lacked explicit communication to all workers. In addition, to improve group engagement, good access to information networks (i.e., trading in local markets) provides better understanding of implemented policies and regulations, which also improves Rule of Law and Corporate Ethics ratings [19]. In Mexico, there are emerging roundtables creating policy incentives for sustainable cattle [37]. These roundtables are a form of a non-state market-driven governance system that includes the creation of working groups that address specific plans and actions towards sustainable development and that can generate opportunities for farms to improve in these themes.

For Environmental Integrity dimension, Land is a relevant SAFA topic for smallholders due to the importance of implementation of practices to preserve soil fertility [36]. When sustainable practices such as manure fertilization or crop rotation, are absent low SAFA ratings occur [19,38]. In contrast, as observed in NS and IS farms, the presence of trees and shrubs granted them better ratings, as they contribute to better soil quality by increasing carbon storage, nitrogen fixation and presence of organic material [6,9]. Furthermore, the presence of tree coverage and diversification of activities, which are common in silvopastoral systems, is related to improved biodiversity by the preservation of endemic flora and fauna [8].

Low ratings in Atmosphere theme are related to the fact that farmers ignore the role of air as an ecosystem service. The absence of appropriate tools to measure actual pollution values generated by units’ activities [13] impedes the creation of awareness through mass media, extension and social networks, which would increase the acceptance of strategies to offset climate change [39]. Knowledge can influence air quality as integration of practices such as manure treatment, mixed-crop systems and feeding cattle with agricultural waste provide better atmosphere performance [19].

Similarly, no clear goals to decrease water pollution or increase water efficiency were present in the units. Education on sustainability is vital to persuade farmers to finance water preservation strategies, as well as reuse and waste disposition systems and management of recycled materials. The possibility to invest in strategies to reduce water waste and pollution improves water ratings [19], and as farms in this study had good investment ratings, water improvement is possible if farmers are properly educated on environmental issues and their impact on their business.

Animal Welfare best and good ratings are obtained when prevention rather than use of veterinary medicines is favored [13] and when animals’ health records, vaccinations and attendance to animal welfare workshops are implemented [19]. Silvopastoral systems are widely recognized for improving welfare and behavior, as tree shade reduces heat stress, improves body condition, reduce the incidence of health problems and favors the presence of affiliative behaviors [8]; nevertheless, as farmers understood the importance of animals’ well-being for farm development, similar ratings were achieved for all systems.

It is worth mentioning that the Environmental Integrity dimension can be greatly supported by the use of digital and scientific tools to measure indicators, such as physical, chemical, bacteriological and microscopic testing of water quality, or air quality monitoring data to determine the amounts of air pollutants emitted by a given system. The farms tested in our study lacked the tools to perform such tests, therefore generating the exclusion of these subthemes; hence the inclusion of these indicators, whenever possible, can strengthen the evaluation of these dimension.

For Economic Resilience, ratings were mainly associated to a local market economy, to the existence of investment goals and to production diversification. In this regard, silvopastoral systems are known for bringing diversification to farms, which in turn creates greater economic security for producers [40,41] and it was expected for silvopastoral systems to be positively rated in all cases for this theme. However, Vulnerability ratings also a reflect factors independent of type of system, such as the ability to identify potential risk for business and the actions taken to avoid such hazards, such as having alternative feed for the dry season and health plans in paddocks to prevent disease [19]. Therefore, it is important that farmers also have enough silvopastoral knowledge to put their benefits into practice.

Lastly, Product Quality and Information, related to the product (calves), was rated as moderate for lacking traceability. It has been mentioned that smaller operations tend to dismiss cow-calf identification systems because farmers are able to remember characteristics of individual cattle [42], which is applicable to farms in the Mexican tropics. Likewise, the National Individual Cattle Identification System (SINIIGA - SINIDA) was not meant to be in full action until 2017, meaning that many small producers in the country did not identified their calves before that year [43,44], making product information one of the subthemes excluded in this study. After 2017, new traceability conditions will allow better rating for this theme.

For the dimension Social Well-Being, low ratings in Decent Livelihood relate to the limited support workers receive in terms of training and fair salary as observed in other cattle smallholders, which rated moderate when no training opportunities and no scores for overtime payment were present [19]. Additionally, a comparison of silvopastoral systems engaged in participatory research (IC) or just traditionally managed (TC) using the SAFA smallholders’ app found limited ratings on questions related to Decent Livelihood, such as the lack of personal protective equipment in IC and TC and the insufficient training provided in TC [20]. Thus, it is important that the intensification of silvopastoral systems in Mexico consider the inclusion of appropriate income, training and safety.

The theme Human Safety and Health is an issue that has received little attention in Latin America, and it has been established that training an education in this specific area can reduce occupational injuries and diseases among workers [45], emphasizing the need for training in all areas. For the theme Labor Rights, none of the farms had full compliance of benefits for their employees, similar to results obtained previously, where this theme was considered a matter of simply compliance for smallholders rather than an important topic [36]. For farms where salary is lower than the norm or family is involved with no payment, Labor Rights were rated as moderate or even unacceptable [19]. Hence, it is important for workers receive education to fully understand and assimilate the importance of their rights, in order to demand improvements on their benefits, and that farm owners are required to provide such knowledge.

Best ratings in Equity relate to no workplace discrimination and the fulfilment of tasks according to agreements [19]. As silvopastoral practices come from cultural practices, Cultural Diversity and silvopastoralism are considered as complementary [46], which could influence the best ratings in all NS farms, where the lack of intensifications maintains traditional values. However, the presence of good-rated MS and IS indicate that other elements, such as the mere presence of indigenous groups in the farm, improves Cultural Diversity [19]. Fair Trading was not reflected in the ratings since cattle prices are set by buyers based on subjective determinations such as weight guessing [19].

The sustainability performance calculated in this study (Table 2) suggests that, under the SAFA framework, NS farms are the most sustainable systems in the Mexican tropics, possibly due to the positive effects on the themes Participation, Land, Biodiversity and Cultural Diversity. However, it is worth mentioning that, in this study, the irrelevance of indicators was managed with subthemes exclusion which in some cases resulted in poor ratings due to the lack of methods to obtain objective measures, as observed for the theme Atmosphere where the subtheme air quality was excluded because measuring standard pollution values in farms was unfeasible. Despite these challenges, the link between system traits and sustainability performance has been previously observed while using the SAFA framework for the evaluation of cattle smallholders [19].

This is the first time SAFA is used to compare silvopastoral systems and monocultures in tropical Mexico. The better ratings of NS systems compared to IS in this study contradict previous findings, where intensified silvopastoral systems in Brazil were better rated than silvopastoral traditional systems when their sustainability performance was evaluated using the SAFA smallholders’ app [20]. The SAFA Smallholders App reduces the original 116 indicators to 44 and works with three ratings (good, limited and unacceptable) [47]. Despite these differences, traditional and intensive silvopastoral systems were categorized as important options for sustainable development when the SAFA app was used, since they had, respectively, 65% and 86% of indicator questions evaluated as good [20]. These results are similar to the percentages encountered in this study for positive valuations of NS and IS farms (Table 2). As both SAFA Framework and SAFA Smallholders App claim to be adequate to evaluate small-scale producers [27,47], further studies need to compare the results obtained with both systems when evaluating silvopastoral farms. This comparison is important, as it has been recognized that the SAFA Smallholders App, even when adjusted from the SAFA Framework to evaluate key sustainability components, can only provide a general picture of sustainability compared to the SAFA Framework, which generates a deeper interpretation of indicators, such as those related to farmers adaptation to climate change, or the time invested in farm activities [14]. Therefore, it is possible that the differences found in other studies assessing systems similar to the ones evaluated here are merely a result of applying a useful, yet less comprehensive SAFA approach.

## 5. Conclusions

This is the first study undertaking the comparison of NS, IS and MS with the use of the SAFA framework in the Mexican tropics’ livestock production sector. This study proved to be helpful to identify differences between systems, create awareness among producers in relation to sustainable management and identify those areas that need to be improved to achieve holistic sustainability goals. Furthermore, the information generated by this study provides useful information on the approach of assessing trade-offs and synergies by using protocols such as the SAFA framework. Future research focusing on developing objective sustainability scoring will be useful for small producers to work on an incentive program agenda, and for more efficient policies on sustainable livestock protein production.

Overall, our main hypothesis was proven, as NS and IS had better sustainable performances than MS. This study identified areas of improvement for a more sustainable performance in cattle farming in Yucatán and possibly for other similar tropical settings in Latin America. Nonetheless, although the SAFA framework is a suitable multicriteria tool to compare the sustainability performance of NS, IS and MS, there are limitations that need to be considered in further studies to properly address the complexity of silvopastoral systems, such as landscape composition and functional complexity, heat stress management in animals and the botanical composition of paddocks. Such improvements will contribute to the final purpose of this kind of research, which is to identify sustainability criteria that allows for the creation of policies and financial incentives aimed to motivate cattle producers to transition to silvopastoral systems, which are a feasible way to foster environmentally friendly cattle production in the Mexican tropics.

## Figures and Tables

**Table 1:** Definitions of Sustainability Assessment for Food and Agriculture (SAFA) dimensions and themes (FAO 2014b).

Dimensions	Themes	Number of Indicators
**Good Governance**: participation of stakeholders and ability to resolve conflicts in relation to other parties affected by farm activities.	**Corporate Ethics**: effective implementation and verification of explicit, public and accessible sustainability objectives	19
	**Accountability**: appropriate corporate behavior responsibility and regular, transparent and public reporting of sustainable performance	
	**Participation**: involvement and identification and invitation to the decision-making process of all interested parties affected by the farm	
	**Rule of Law**: commitment with justice and legitimacy with explicit rejection of corruption, extortion and the use of resources that are legally disputed. Protection of the environment and vulnerable workers using robust and applicable codes and laws	
	**Holistic Management**: Production and supply appropriately managed considering all sustainability dimensions	
**Environmental Integrity**: preservation of the essential live sustenance systems for human beings while minimizing negative impacts and improving positive environmental outcomes	**Atmosphere**: actions that farms undertake to reduce as much as possible the liberation of greenhouse gases and those air contaminants that could threaten ecosystem health	52
	**Water**: use of freshwater extraction methodologies that do not interfere with natural cycles, create pollution or have a negative impact on ecosystem health	
	**Land**: prevention of arable land or grasslands loss by the implementation of practices to preserve and improve soil fertility	
	**Biodiversity**: implementation of sustainable management to promote the conservation of flora and fauna	
	**Materials and Energy**: use of renewable energy sources and methods to dispose, recycle or reuse waste materials to reduce ecosystem damage	
	**Animal Welfare**: presence of conditions that allow animals to express their natural behaviour, as well as the absence of thirst, hunger, pain distress and sickness	
**Economic Resilience**: implementing measures to promote the recovery capacity of a system when adversities and eventualities arise	**Investment**: financial endowment of capital goods, human resources or ecosystems, either internally, in associated communities or long-term investment for sustainable development	26
	**Vulnerability**: resilience of production, supply and commercialization in terms of environmental, economic and social challenges	
	**Product Quality and information**: abstaining from generating any kind of pollution that could produce harmful substances. Ability to have product traceability	
	**Local Economy**: contributions that farms make to local economic development	
**Social Well-Being**: satisfaction of basic human needs and provision of rights to satisfy aspirations for a better life	**Decent Livelihood**: provision of capacities and activities that increase the sustenance and the security of the personnel and the neighboring community	19
	**Fair Trading Practices**: presence of fair-trade practices for the buyers and sellers with prices reflecting the true cost of the maintenance and regeneration of an ecological system including the welfare of workers	
	**Labor Rights**: employment compliancy with national and international law,	
	**Equity**: strict pursue of equity and provision of support to vulnerable groups	
	**Human Safety and Health**: provision of a healthy, hygienic and safe work environment with the necessary elements to satisfy human needs (clean water, food, proper facilities, etc.)	
	**Cultural Diversity**: respect of intellectual property and rights of indigenous groups	

**Table 2:** Soil characteristic evaluated per SAFA indicator for the subtheme “Soil Quality”.

Safa Indicator for Soil Quality	Soil Characteristic Evaluated
Soil Physical Structure	Texture and percentage of organic matter
Soil Chemical Quality	NO3–NO4 relation and total organic phosphorus content
Soil Biological Quality	NO3–NO4 relation and total organic carbon content
Soil Organic Matter	Total organic carbon content

**Table 3:** Evaluation of sustainability performance in farms ^1^.

Farm	Valuations	Safa Category
Roble NS1	+67% −14%	best
		good
Kampepem IS2	+62% −19%	best
		moderate
Santa Teresa NS2	+57% −14%	best
		good
Xhopel NS3	+48% −14%	good
		good
UADY M2	+48% −14%	good
		good
Kakalnah IS1	+48% −19%	good
		moderate
Escalera M3	+33% −10%	moderate
		good
Las Golondrinas IS3	+33% −14%	moderate
		good
Ramonal M1	+24% −38%	limited
		unacceptable

^1^ NS farms had best and good ratings for positive and negative valuations. IS farms had lower sustainability performance, with positive ratings between best and moderate and negative ratings between good and moderate. In the MS farms, ratings were highly variable, and they presented the worst rated farm (M1), with limited positive valuations and unacceptable negative valuations.
